# The Sense of Agency as Tracking Control

**DOI:** 10.1371/journal.pone.0163892

**Published:** 2016-10-14

**Authors:** Emilie A. Caspar, Andrea Desantis, Zoltan Dienes, Axel Cleeremans, Patrick Haggard

**Affiliations:** 1 Consciousness, Cognition and Computation Group (CO3), Center for Research in Cognition & Neurosciences (CRCN), ULB Neuroscience Institute (UNI), Université libre de Bruxelles (ULB), Brussels, Belgium; 2 Institute of Cognitive Neuroscience, University College London (UCL), London, United Kingdom; 3 University of Sussex, School of psychology, Brighton, United Kingdom; Ludwig-Maximilians-Universität München, GERMANY

## Abstract

Does sense of agency (SoA) arise merely from action-outcome associations, or does an additional real-time process track each step along the chain? Tracking control predicts that deviant intermediate steps between action and outcome should reduce SoA. In two experiments, participants learned mappings between two finger actions and two tones. In later test blocks, actions triggered a robot hand moving either the same or a different finger, and also triggered tones, which were congruent or incongruent with the mapping. The perceived delay between actions and tones gave a proxy measure for SoA. Action-tone binding was stronger for congruent than incongruent tones, but only when the robot movement was also congruent. Congruent tones also had reduced N1 amplitudes, but again only when the robot movement was congruent. We suggest that SoA partly depends on a real-time tracking control mechanism, since deviant intermediate action of the robot reduced SoA over the tone.

## Introduction

Healthy adults generally have a strong feeling of voluntarily control over their actions. In particular, they are able to initiate and endogenously control actions to achieve desired goals. This process involves a «sense of agency» (SoA), i.e., a subjective feeling of control over one’s own actions and their outcomes [1].

Human SoA can adapt to rather complex causal chains mediating between action and outcome. This crucial capacity underlies the highly developed technological, automation and interpersonal chains on which modern society depends [2]. For example, someone may feel a strong sense of agency when clicking on a website to get a pizza delivered, yet the intermediate steps between click and consumption are legion and complex. Even using a simple tool involves the same processes of mediation between action and outcome. For example, someone who wants to pick and eat an apple from a tree may have to use several intermediate steps, such a ladder to approach it, a gripper to grasp it, and a basket to catch it. The cognitive mechanisms that underpin SoA in the context of such extended causal chains could potentially be understood by identifying where breaks may occur along the intention-action-outcome chain [3]. One influential view suggests that SoA is a retrospective inference based on the coincidence of action, outcome, and prior thought [4]. On this view, unexpected or deviant mediation between action and outcome should not alter SoA as long as the match between prior intentions and final outcomes is preserved (see [3] for a review). However, this cannot explain why humans do not always feel control when intentions and outcomes merely coincide. Alternatively, accounts of ‘tracking control’ [5] suggest that SoA tracks the intermediate steps by which an action achieves an outcome, thus avoiding spurious correlations. Indeed, if a failure occurs during the planed causal chain, but the final and expected outcome is finally reached, people may attribute this success to chance rather than to themselves. Thus, tracking control theories assume that altered causal chains should reduce sense of responsibility, even when the outcome is as intended.

In a previous study, we observed that conflicting feedback from a robot regarding what a participant actually did could reduce SoA, even if the final outcome of action was as intended [6]. This highlights the importance of correct, real-time predictions about means, as well as ends, for the sense of agency.

However, to our knowledge, the hypothesis that SoA depends on a tracking control process has not yet been directly addressed. Importantly, testing this hypothesis requires a more complex causal chain than the simple instrumental action-outcome relations usually considered in previous studies. It also requires an implicit measure of agency. Indeed, explicit questions of agency over ends would presumably underestimate any effects of means, and the importance of tracking the intermediate steps in the causal chain, because of the strong tendency to *reconstruct* narratives of successful agency once final outcomes are known [7]. Instead, we used an implicit measure of agency, based on the perceived interval between action and outcome. This interval is perceived as shorter for intentional actions than for unintended actions [8], providing a useful proxy for SoA. Here, participants made one of two actions to achieve an associated outcome tone. Their movements also triggered a task-irrelevant robotic hand whose movement was either congruent or incongruent with the participant’s action. The robot movement was independent of the action-tone mapping.

If SoA depends on a tracking control process, observing a non-homologous robot movement should automatically lead to a re-computation of the expected tone, thus reducing the feeling of control, even if the tone later turns out to be homologous with the original learning. In a second experiment, we sought to investigate the brain mechanisms responsible for the contribution of tracking control to SoA, using event-related potentials.

## Experiment 1

### Method

#### Participants

A total of 21 right-handed and naïve participants were recruited. The sample size was based on previous studies of agency (e.g. [9]). Participants received £7.50 for their participation. The following exclusion criteria were decided in advance of the experiment: failure to produce temporal intervals covarying monotonically with actual action-tone interval, or failure to follow instructions. To identify participants, whom the action-tone intervals did not gradually increase with action-tone intervals, we performed linear trend analysis with contrast coefficients -1, 0, 1 for the three delays. Five participants were excluded due to a non-significant linear trend analysis. Of the 16 remaining participants, 6 were males. The mean age was 22.72 (SD = 7.09). All participants provided written informed consent prior to the experiment. The study was approved by the local ethical procedures of the Institute of Cognitive Neuroscience at University College London.

#### Procedure & Material

Participants sat with their right hand below a table, while a right human-like robotic hand was placed in full view on the table above their hand (see [10–11] for more information about the robotic hand). One keyboard was provided for the participant, and one for the robot. The fingers of the robotic hand were placed so as to visually convey the impression of pressing exactly the same key as the participant. Participants controlled this robotic hand by wearing a glove fitted with sensors. As a result, when they moved a finger, the robotic hand moved at the same time.

Participants were first asked to learn how to produce sequences of four tones, by making appropriate voluntary actions. The tone sequences were composed of 2400Hz and 2600Hz tones, in a different random order for each block. Participants could press either the ‘F’ key with the index finger, or the ‘H’ key with the ring finger. The mapping between keys and tones was counterbalanced across participants. Participants were instructed to listen to the four-sound sequence, and then to try to reproduce it. In this phase, the robot movements were always homologous with the participants’ movements. This task aimed at ensuring that participants correctly learned the correct mapping between keys and sounds, and so grasped the normal congruent chain of events. The delay between key press and the sound was randomly drawn from the values 300, 500, 700 ms. Learning the sequence of four tones was not relevant to our hypotheses, but provided a suitable learning challenge in which participants could engage, and which would ensure that they learned the action-tone mappings.

**Fig 1:**
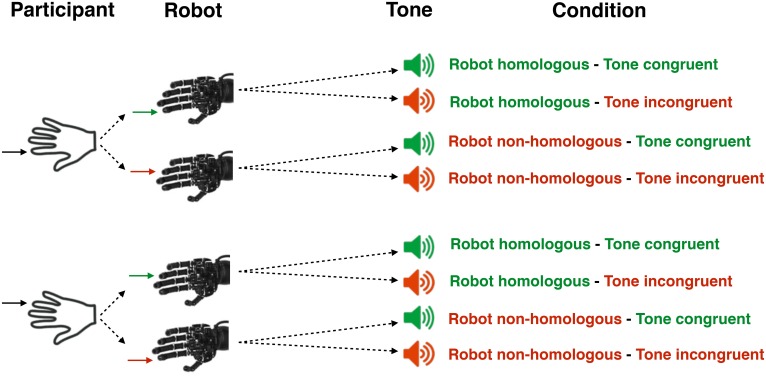
graphical representation of the four conditions.

In the test phase, participants were told to freely choose between pressing either the ‘F’ key or the ‘H’ key whenever they wanted but to try to produce the same amount of the two movements in the long run. The participants’ task was to estimate the delay between the keypress and the tone by absolute numerical estimation. Participants were instructed that the delay varied randomly on a trial-by-trial basis, and never exceeded 1000 ms. In reality, only three delays were used, i.e. 300, 500 and 700 ms. In this task, there were four experimental conditions (Fig 1). In the *robot homologous-tone congruent* condition, participants pressed one of the two keys, the robotic hand immediately reproduced exactly the same key press and the tone was congruent with the action, according to the relation presented in the association phase. The delay between the participant’s action and robot action was calculated as 14 ms (SD = 17.16, see Caspar et al., 2015 for a description of action-robot delays). In the *robot homologous -tone incongruent* condition, participants pressed one of the two keys, the robotic hand reproduced exactly the same key press with the same finger at the same time, but the tone was not the expected one (e.g. if ‘H’ was associated with a 400Hz tone in the association phase, ‘H’ was associated with a 600Hz tone in this condition). In the *robot non-homologous -tone congruent* condition, participants pressed one of the two keys, but the robotic hand moved the other finger. However, the tone was as learned during the association phase. Finally, in the *robot non-homologous—tone incongruent* condition, participants pressed one of the two keys, but the robotic hand moved the other finger and the resulted sound was not the one learned. To sum up, the experimental design involved a random and equiprobable occurrence of robot actions and tones in a factorial design. These two event types were statistically independent from each other.

**Fig 2:**
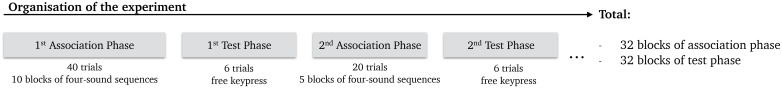
Schematic representation of the association phases and test phases.

Each test phase was composed of 6 free trials (= 6 free keypresses), all drawn from the same condition. We did not mix the four conditions within blocks of the test phase because the robot software did not allow rapid changes of the mapping between participant fingers and robot fingers. Thus, the first block of the experiment was composed of ten short blocks of four-sound sequences (= 40 trials) of an association phase in order for participants to learn all action-outcome mappings. Then, participants alternated between six free-choice trials in a test phase (2 trials for each delay, randomly presented) and five short blocks each composed of four-sound sequences in the association phase (Fig 2). In total, there were 192 trials in test phases (32 blocks x 6 trials) and 660 trials in association phases (31 blocks x 5 sequences x 4 sounds = 620 + 1 block of 40 trials). Thus, the ratio between association trials and test trials was 3.43. To summarize, participants performed 32 blocks of association and test trials, resulting in a total of 812 voluntary actions.

### Results

#### Behavioral Results

We compared the percentage of correct reproductions in learning phases occurring after each experimental condition in the test phases (Figure B in S1 File). We did not see evidences that they extinguish during the test phase the learning about action-tone mapping that had been established in the previous learning phase. This suggests that the ratio used in Experiment 1 (6 test trials for 20 learning trials) was sufficient.

#### Intentional binding

Intentional binding refers to a perceptual compression of the interval between voluntary action and outcome. In our interval estimation task, a condition with lower interval estimates would be said to show greater intentional binding than a condition with higher interval estimates. Effects were tested using Bayes factors, B, to assess strength of evidence; p-values are also reported so readers can in addition assess significance (see [12–13]). B-values of 3 or above indicate substantial evidence for the alternative rather than the null hypothesis. B-values of 1/3 or below substantial evidence for the null rather than alternative hypothesis. Intermediate values mean the data are insensitive for distinguishing the alternative and null hypotheses. Our central predictions involved the effect of means-ends consistency on feelings of agency as measured by intentional binding. B_H(0,432)_ refers to a Bayes factor used to test the alternative hypothesis that there is a difference between conditions, represented as a half-normal with a SD of 432 ms, against H0, the hypothesis of no difference. Following Dienes (2014), when a roughly expected effect size can be specified, it is used as the SD of a half-normal. The overall mean interval estimates for the present data (i.e. 432 ms) was taken as the order of magnitude difference expected for specific group comparisons.

A Robot action (homologous, non-homologous) x Tone congruence (congruent, incongruent) x Delay (300, 500, 700) repeated-measures ANOVA was conducted on participants’ mean interval judgment. There was evidence for no main effect of Robot action (F(1,15) = 0.023, *p* > .8, η^2^_***partial***_ = .002, B_H(0,432)_ = 0.20). There was evidence for a main effect of Tone congruence (F(1,15) = 9.929, *p* = .007, η^2^_***partial***_ = .398, B_H(0,432)_ = 22.28), with shorter mean interval estimation for congruent tones (409.381, 95% CI: 343.671–475.090) than for the incongruent tones (455.66, 95% CI: 377.674–533.658). Unsurprisingly, there was a significant main effect of Action-Tone Delay (F(2,30) = 54.170, *p* < .001, η^2^_***partial***_ = .783), which was not further investigated.

**Fig 3:**
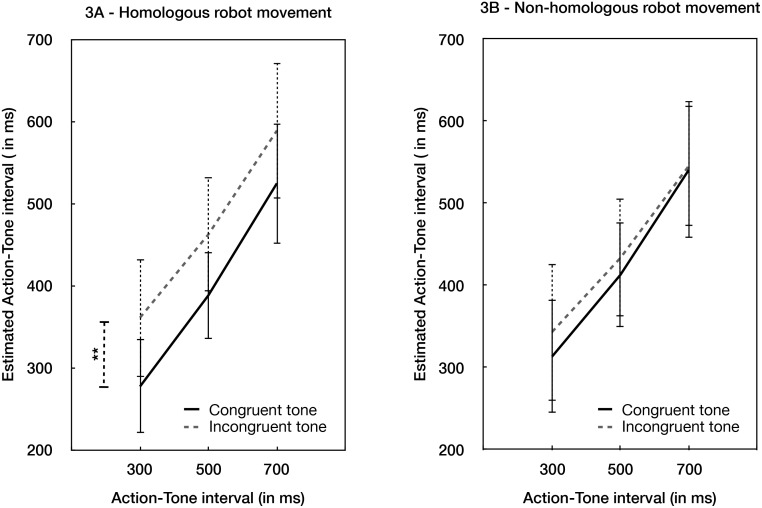
Behavioural Results. (A) Interval judgment estimation in the two experimental conditions in which the robot movement was homologous with the participant’s movement. (B) Interval judgment estimation in the two experimental conditions in which the robot movement was non-homologous with the participant’s movement. ** indicates a significant difference (two-tailed p < .01). Error bars refer to standard errors. All the tests were two-tailed.

There was an interaction between Robot action and Tone congruence, F(1,15) = 7.398, *p* < .020, η^2^_***partial***_ = .330, B_H(0,432)_ = 6.70 (Fig 3A & 3B). Analyses of simple effects revealed that when the robot action was homologous and followed the normal causal chain, then congruent tones produced more binding (396.54, 95% CI: 334.646–458.434) than incongruent tones (443.87, 95% CI: 367.06–520.67), t(15) = -2.959, *p* = .01, Cohen’s d = .739, B_H(0,432)_ = 18.82). However, when the robot action did not follow the expected causal chain, there was substantial evidence for no effect of Tone congruence on binding (t(15) = -1.186, *p* > .1, B_H(0432)_ = 0.13). That is, effects of congruent outcomes were found only when the intermediate steps in the chain were also homologous. We also investigated the effect of the robot’s movement, holding the tone outcome constant. When the tone was congruent with the action, there was evidence for no effect of the robot’s movement (t(15) = 1.492, *p* >.1, B_H(0,432)_ = 0.26). When the tone was incongruent, the effect of the robot’s movement was irrelevant for the binding (t(15) = 1.724, *p* > .1, B_H(0,432)_ = 0.34). Other two-way interactions, and the triple interaction did not provide evidence for further effects (all B’s < 3, *ps* > .4).

### Discussion

The interaction between tone congruence and the robot action shows that the contribution of outcome expectation was diminished when there was conflicting visual feedback from the robot regarding what the participant had actually done. Thus, if the robot produces a deviant intermediate step in the causal chain, then the SoA is disrupted. In particular, the stronger binding for congruent rather than incongruent trials depends on a consistent intermediate chain between means and ends.

Strong ideomotor theories of SoA propose that intentional actions are represented in the brain in terms of their outcomes. For instance, the theory of apparent mental causation [14] suggests that no conscious access is possible to the inner workings of the motor system in action generation. Thus, the sense of agency is simply inferred from the combination of prior thought and outcomes, without tracking intermediate steps. In general, ideomotor theories would tend to view intermediate steps between prior thought and outcome as irrelevant to the representation of intentional action (e.g. [15–16]). On one popular view, the intermediate steps of the causal chain between thought actions would be inferred *retrospectively* once the perceptual concordance between an action’s idea and an action’s effect has been noticed, and then re-inserted into the conscious narrative of action [17]. A strong version of that view predicts that unusual intermediate steps in the causal chain should not reduce SoA. However, our data showed a clear reduction in SoA in the *robot non-* homologous *-tone congruent* condition, those results support the alternative hypothesis of a real-time “tracking control” process, which computes SoA during the development of action itself.

However, because our interval estimate judgements were obtained only after the outcome, they cannot conclusively rule out a reconstructive component of SoA. Therefore, in a second experiment, we looked for real-time neural correlates of this “means-mediated SoA”. Specifically, the tracking control hypothesis predicts that the robot hand movement should evoke an online neural activity that differs between homologous and non-homologous robot movements. For example, the homologue/non-homologue of the robot movement should influence the neural processing of the subsequent tone, according to a tracking control hypothesis. In contrast, the ideomotor hypothesis predicts that intermediate events prior to the final, distal outcome should have no effect on processing of that outcome.

Sensory attenuation of evoked potentials provides a suitable neural marker. Previous studies have shown a sensory attenuation of early brain components evoked by a congruent, compared to incongruent, outcome of action (e.g. [18–20]), but non-homology of means has not been examined before. Based on our assumption that SoA depends on a successful tracking control process, a non-homologous robot action should not reduce the degree of sensory attenuation after the tone, even if the outcome is congruent with participant’s action. In contrast, a fully congruent causal chain, in which both robot action and tone are congruent with the participant’s action, should produce the strongest sensory attenuation.

## Experiment 2

### Method

#### Participants

A total of 18 right-handed and naïve participants were recruited. Participants received 20€ for their participation. The exclusion criteria were as in Experiment 1. To identify participants, whom the action-tone intervals did not gradually increase with action-tone intervals, we contrasted the two action-outcome delays (see later). Two participants were excluded due to lack of evidence of a significant relation between action and perceived delay. In addition, the electrophysiological signal of one participant exceeded the mean number of rejected trials by two standard deviations and they were therefore excluded. Of the 15 remaining participants, 8 were males. The mean age was 24.73 (SD = 3.95). All participants provided written informed consent prior to the experiment. The study was approved by the local ethical committee of the Faculty of Psychological Science and Education of the Université Libre de Bruxelles (ULB).

#### Procedure & Material

The procedure was globally the same as in Experiment 1. However, a few modifications were made to adapt the design to EEG recordings. Again, association phases and test phases alternated. In the association phase, participants had to repeat a single sequence of 4 tones with a full congruent chain of events. In the test phases, participants only performed one free trial (against six in Experiment 1), because technical difficulties did not allow the robot to switch rapidly from homologous to non-homologous actions. With this procedure, the ratio between learning trials and test trials was slightly higher than Experiment 1 (= > 4 sounds to reproduce in the association phase /1 test trial = 4). In order to have a good baseline for the test phases, a grey screen was displayed with the words “*please remain still*” during 1.5 seconds at the beginning of the test phase and during 1.5 seconds after the tone. Participants were instructed to press whichever key they wanted in the test phase, but not to press when the screen displayed those words. Three practice blocks were administered before the main experiment. In total, participants performed 200 blocks (1 block = 1 association phase + 1 test phase). Each experimental condition was randomly presented in 50 test phases.

Participants were instructed that the delay varied randomly on a trial-by-trial basis, and was between 500 ms and 1500 ms. In reality, only two delays were used, 800 and 1100 ms. We used longer action-tone intervals than those used in Experiment 1 to allow a sufficient EEG epoch for each event.

#### Electrophysiological recordings

Cerebral activity was recorded using a 64-channels electrode cap with the ActiveTwo system (BioSemi) and data were analysed using Fieldtrip software [21]. The activities from left and right mastoids and from horizontal and vertical eye movements were also recorded. Amplified voltages were sample at 2048 Hz. Data were referenced to the average signal of the mastoids and filtered (low-pass at 50 Hz and high-pass at 0.01 Hz).

### Results

#### Behavioral Results

Again, we compared the percentage of correct reproductions in learning phases occurring after each experimental condition in the test phases (Figure B in S1 File). We did not observe any statistical differences between learning blocks performed after each experimental condition occurring in the test phases.

#### Intentional binding

Experiment 1 found effects of intermediate causal chains amounting to around 50 ms. Thus, for experiment 2, H1 was modelled as a half-normal with an SD of 50 ms. The data were tested for normality with a Shapiro–Wilk test (*p* > .05). A Robot action (homologous, non-homologous) x Tone congruence (congruent, incongruent) x Delay (800, 1 100) repeated-measures ANOVA was conducted on participant’s mean interval judgment. There was no evidence for a main effect of Robot action homologue/non-homologue (F(1,14) = 1,580, *p* > .2, η^2^_***partial***_ = .101, B_H(0,50)_ = 1.01). The evidence favoured an effect of Tone congruence/incongruence rather than H0, (F(1,14) = 4.198, *p* = .06, η^2^_***partial***_ = .231, B_H(0,50)_ = 3.67), with shorter mean interval estimation for congruent tones (900.737, 95% CI: 824.04–977.43) than for incongruent tones (924.02, 95% CI: 851.70–996.33). Again as expected, there was a significant main effect of Delay (F(2,14) = 44.975, *p* < .001, η^2^_***partial***_ = .763), which was not analysed further.

**Fig 4:**
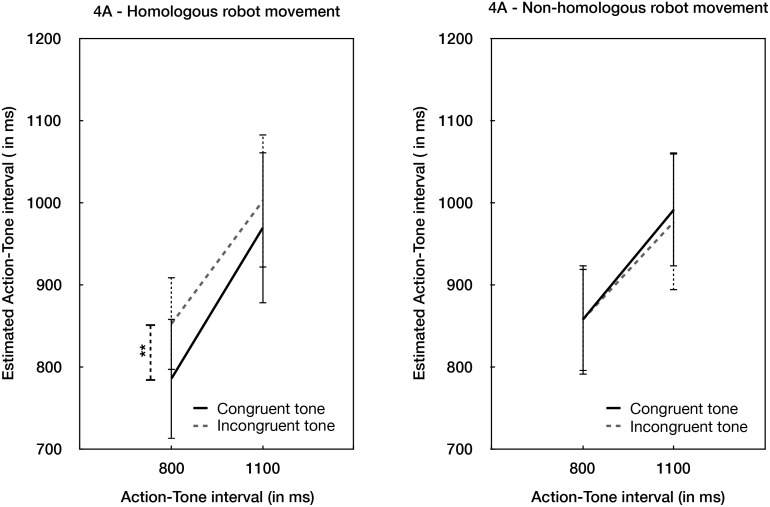
Behavioural results. (A) Interval judgment estimation in the two experimental conditions in which the robot movement was homologous with the participant’s movement. (B) Interval judgment estimation in the two experimental conditions in which the robot movement was non-homologous with the participant’s movement. ** indicates a significant difference (two-tailed *p* < .01). Error bars refer to standard errors. All the tests were two-tailed.

As in Experiment 1, there was an interaction between Robot action and Tone congruence, F(1,14) = 10.19, *p* = .007, η^2^_***partial***_ = .421, B_H(0,50)_ = 165.79 (Fig 4A & 4B). Analyses of simple effects revealed that when the robot action was homologous with the participant’s action and followed the normal causal chain, the congruency of the tone produced a higher binding (878.29, 95% CI: 792.41–964.17) than when the tone was incongruent (930.45, 95% CI: 860.39–1000.51), t(14) = -3.499, *p* = .004, Cohen’s d = .903, B_H(0,50)_ = 536.40). However, when the robot action did not follow the expected causal chain, there was marginal evidence that the congruency of the tone was irrelevant to binding (t(14) = .396, *p* > .6, B_H(0,50)_ = 0.36). As in Experiment 1, we investigated the effect of the robot’s movement, holding the tone outcome constant. When the tone was congruent with the action, there was a significant effect of the robot’s movement, with non-homologous robot movements producing higher interval estimates (i.e., less binding) than homologous robot movements (t(14) = -2.582, *p* = .022, η^2^_**partial**_ = 0.129, B_H(0,50)_ = 12.76). When the tone was incongruent, the effect of the robot’s movement was irrelevant for binding (*p* > .3, B_H(0,50)_ = 0.67). Other two-way interactions, and the triple interaction were non-significant (all B’s < 3, *p*s > .1).

#### Event-related potentials

ERPs were extracted for two events in each trial. The first was the combined event of the participant’s action and quasi-simultaneous robot action. The second event analysed was the tone caused by the action. For Bayes factors, H1 was modelled using the average peak of the auditory N1 as information about the scale of the effect that could be expected (for each participant, all trials were averaged for Fz, FCz, and Cz; and then an average taken over participants). As manipulations rarely eliminate a component, the average was treated as a rough maximum effect that could be expected, and H1 thus modelled as a half-normal distribution with the standard deviation set to half the average peak. The half-normal was used because the theory made directional predictions [13].

#### Brain potentials associated with participants’ and robot action

ERPs were time-locked to the onset of the key press of the participant with a time window from -700 to 600 ms. Baseline correction was performed for the time from -700 to -500. Epochs containing artefacts such as blinks were rejected based on visual inspection. On average, 78 (mean = 22.13) of the 100 trials were artefact-free (SD = 9.69). The remaining artefact-free trials were averaged separately in accordance with the two different conditions associated with the participant’s action: a homologous robot movement and a non-homologous robot movement.

A clear component occurring after the participant’s key press was identifiable from the grand averages, that is, the visual N1, a brain component elicited by visual stimuli. Indeed, participants were instructed to look towards the robotic hand during the whole experiment. The robot action can thus be considered as a visual stimulus available in the centro-right visual field of participants.

**Fig 5:**
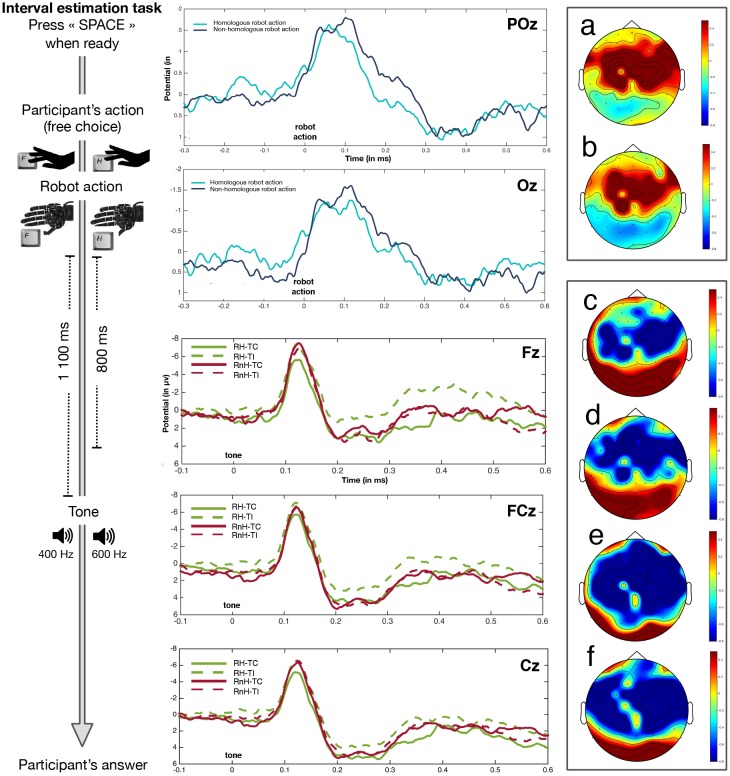
Electrophysiological results. On POz and Oz, the light blue displays the visual N1 associated with a homologous robot movement, while the dark blue displays the visual N1 associated with a non-homologous robot movement. On Fz, FCz and Cz, the full green line displays brain potentials associated with the robot homologous—tone congruent condition (RH–TC), the dotted green line displays brain potentials associated with the robot homologous—tone incongruent condition (RH-TI), the full red line displays brain potentials associated with the robot non-homologous—tone congruent condition (RnH-TC), and the dotted red line displays brain potentials associated with the robot non-homologous—tone incongruent condition (RnH-TI). (A) Topographical representation associated with homologous robot movement. (B) Topographical representation associated with non-homologous robot movement. (C) Topographical representation associated with RH-TC. (D) Topographical representation associated with RH-TI. (E) Topographical representation associated with RnH-TC. (F) Topographical representation associated with RnH-TI.

Analysis of the visual N1 was measured as mean amplitude across Oz and POz. We determined the visual N1 amplitude with the more negative peak within the 30–170 time window. There was an evidence as to whether the visual N1 was more negative after a non-homologous robot movement than after a homologous robot movement (t(14) = -2.954, *p* = .01, Cohen’s d = .762, B_H(0,1.7)_ = 42.69), see Fig 5a and 5b.

#### Brain Potentials associated with the tone

ERPs were time-locked to the tone with a time window ranging from -300 to 600 ms, irrespective of the delay between the action and the tone. Baseline correction was performed for the time from -300 to -200. Windows were determined in order to not overlap with potentials occurring after the robot action. Epochs containing artefacts were rejected based on visual inspection. On average, 38 (mean = 12.66) of the 50 trials were artefact-free (SD = 8.59). The remaining artefact-free trials were averaged separately in accordance with the four different conditions associated with the tone and in accordance with the robot action: robot homologous—tone congruent condition, robot homologous—tone incongruent condition, robot non-homologous—tone congruent condition, and robot non-homologous—tone incongruent condition.

Two components occurring after the tone were identifiable from the grand averages. These were the auditory N1 and the P3, an event-related potential linked to the evaluation of a stimulus [22].

Analysis of the auditory N1 was measured as mean amplitude across Fz, FCz and Cz [18]. We determined the auditory N1 amplitude as the most negative peak within the 90–170 time window. A Robot action (homologous, non-homologous) x Tone congruence (congruent, incongruent) repeated-measures ANOVA revealed a main effect of Robot action, F(1,14) = 11.486, *p* = .004, η^2^_***partial***_ = .451, B_H(0,4)_ = 153.65. The amplitude of the auditory N1 was smaller for tones preceded by a homologous robot movement than for tones preceded by a non-homologous robot movement. There was no main effect of Tone congruence (F(1,14) = 1.029, η^2^_***partial***_ = .068, *p* > .3, B_H(0,4)_ = 0.23). There was no clear evidence one way or the other for the Robot action x Tone congruence interaction (F(1,14) = 3.303, *p* = .09, η^2^_***partial***_ = .191, B_H(0,4)_ = 1.74). Note that the non-significance of the interaction in no way counts *against* our theory predicting it, it merely fails to provide strong evidence for it. Bayes factors for simple effects are not dependent on previously identifying an interaction. As we had made clear predictions about specific conditions, the simple effects could therefore be tested. The auditory N1 amplitude was smaller for congruent than incongruent tones, only when the robot action was homologous with the participant’s action (t(14) = -3.256, *p* = .006, Cohen’s d = .840, B_H(0,4)_ = 94.49). When the robot action was non-homologous, there was substantial evidence for the incongruence of the tone not affecting the auditory N1 amplitude (t(14) = 0.299, *p* > .7, B_H(0,4)_ = 0.16).

Topographical representation of the 90–170 ms time window across the four conditions revealed a higher negativity in the fronto-parietal areas when either the robot was non-homologous (i.e. RnH-TC condition) or the tone was incongruent (RH-TI condition), see Fig 5c, 5d, 5e and 5f. The lower negativity in the robot homologous-tone congruent and robot non-homologous—tone incongruent conditions suggests that a match between the robot action and the tone reduced the conflict over the tone.

For the analysis of the P3, we applied the same procedure. Again, we computed the mean amplitude across Fz, CPz and Cz. The amplitude of the P3a was determined by the mean within the time window of 270–300 ms post stimulus and expressed as μV. A Robot action (homologous, non-homologous) x Tone congruence (congruent, incongruent) repeated-measures ANOVA revealed a main effect of Tone congruence, F(1,14) = 6.435, *p* = .024, η^2^_***partial***_ = .315, B_H(0,1.8)_ = 15.08. The amplitude of the P3 was higher for congruent tones than for incongruent tones. The main effect of Robot action was not sensitive (F(1,14) = 0.887, *p* > .3, η^2^_***partial***_ = .0.60, B_H(0,1.8)_ = 0.60). There was substantial evidence for an interaction between Robot action x Tone congruence (F(1,14) = 4.294, *p* < .06, η^2^_***partial***_ = .235, B_H(0,1.8)_ = 6.15). Paired sample t-tests indicated that the P3 amplitude was higher for congruent tones than for incongruent tones when the robot action was homologous (t(14) = 2.575, *p* = .022, Cohen’s d = .664, B_H(0,1.8)_ = 24.99). When the robot action was not homologous, then the P3 amplitude was not different according to the congruency of the tone (t(14) = 0.604, *p* > .5, B_H(0,1.8)_ = 0.23).

### Discussion

Experiment 2 replicated the behavioural results of Experiment 1, confirming again that the linkage between action and outcome is of importance for binding if the means used to perform the desired outcome are not in conflict with the expected causal chain.

Regarding brain components associated with the participants’ and the robot actions, we found a more negative visual N1 when the robot movement was non-homologous with the participant’s action than when this movement was homologous, suggesting that participants kept their attention on the robot’s actions during this experiment. This is consistent with previous studies showing that the amplitude of the visual N1 increases with attention (e.g. [23]) or conflict [24].

Surprisingly, the P3 amplitude was smaller for incongruent tones than for congruent tones after a homologous robot movement, while P3 amplitude is classically enhanced after unexpected feedback (e.g. [25]). This might be due to a higher probability to get incongruence (75% versus 25%) in test phases.

## General Discussion

In the present paper, we assessed whether SoA could be the result of a cognitive computation that tracks all the steps of a chain of events up to the final outcome. In Experiments 1 and 2, behavioural results showed stronger SoA when an action and its outcome were congruent rather than incongruent, but only when the additional event of the robotic hand movement was also congruent, and not when it was incongruent. Electrophysiological results were similar. The difference in auditory N1 between tones that were congruent or incongruent with the previous action was sensitive to whether the robot action was also congruent or not.

Interestingly, we observed that visual N1 was attenuated when the movement of the robot hand was homologous with participants’ action. Similarly, auditory N1 components were attenuated for congruent tones when the robot action was also homologous. This replicates previous findings observing sensory attenuation of predicted action-effects [18, 19–20, 26–27].

The psychophysiological results are broadly consistent with the behavioural data in both Experiments 1 and 2. Interval estimations showed that the contribution of outcome expectation is diminished when there is a conflicting visual feedback from the robot regarding what the participant actually did. ERP analysis showed that the congruent attenuation of N1 amplitude for congruent tones was similarly abolished when the robot’s movement was non-homologous. Sensory attenuation is widely viewed as a consistent marker of processing of self-generated events [28–29]. Our results therefore suggest that an intermediate step of action processing, provided by the non-homologue movement of the robot hand, leads to a revision of the prediction regarding the outcome. Specifically, when the robot hand moves in a non homologous manner, predictions regarding the tone outcome would be weakened, leading to loss of the sensory attenuation effect. This implies that the prediction of final outcomes is continuously updated during the course of action processing by intermediate steps along the causal chain. Moreover, this updating process must be automatic: in our study, the movement of the robot hand was statistically independent of action and tone. Therefore, although the robot movement was actually irrelevant to predicting the tone outcome, it automatically leads to on-line re-computations of both predictability (as evidenced by N1 attenuation) and of SoA (as evidenced by interval estimates). This continuous, on-line recomputation of agency is consistent with Bratman’s concept of tracking control [5], and inconsistent with strongly reconstructionist accounts of SoA.

The present results suggest an additional element in the basic predictive model. The experience of outcome congruence is necessarily retrospective, since the match can only be made after the actual effects are known. The present study suggests that SoA additionally depends on a continuous “tracking control” process that continuously predicts intermediate steps along each step of the causal chain. A failure of means can lead to a reduced sense of control, as we observed in the present results. This view suggests that many elements in the causal chain are important for SoA, and that they are arranged in a sequence reflecting causal order. If a failure occurs in an early event, this initiates a reduction of the feeling of control for the subsequent events. This conclusion is supported by recent work showing that a feedback about action performance in a Flanker task can recalibrate the prediction of sensory outcomes and reduced SoA [30]. This modification suggests that SoA is based on a real-time cascade of action processing, and not only on retrospective inference from outcomes.

In the present paper, we used Bayes factors to evaluate strength of evidence for either the experimental or the null hypothesis. Evidence strength is not a routine concept in orthodox statistics (e.g. [12–13, 31–37]. Thus, we could evaluate whether a non-significant result reflected evidence for the null hypothesis or not much evidence at all.

Importantly, our results do not indicate that the means (i.e., robotic hand movement) used to achieve a goal are ***more*** important than the ends (i.e., tone) in generating a vivid sense of agency. Rather, our simple effects test suggested that homology of the robotic hand is comparable in importance to the congruency of the tones. In experiment 2, when the tone was congruent with participants’ action, non-homologous robot movements produced higher interval estimates (i.e., less binding) than homologous ones. However, when the tone was incongruent, the robot action was no longer relevant for the binding effect. Therefore, the sense of agency seems to be enhanced by a specific relationship between means and ends, due to an on-line computation that tracks the sequential stages of the causal chain between intentions and outcome. Since our study used an implicit measure based on time perception, and did not require any explicit judgement about agency, we further assume that on-line computations of means-ends relations for sense of agency proceed automatically.

Several previous studies have shown that predictions about external, visible outcomes are more important for agency than internal sensorimotor predictions. For instance, Fourneret and Jeannerod [38] have shown that people make unconscious adjustments to their movements to achieve an external, visually-specified goal. This suggests that external, visual cues may be more important for SoA than internal sensorimotor cues [39]. In our study, the robot hand was salient and visible, whereas the participant’s own hand was obscured, as is normal in agency studies. Boosting the external representation in this way could have increased the contribution of the robotic hand movement to the sense of agency over the outcome, thus explaining the interaction between robot action congruence and tone congruence.

How the participants construed the action of the robotic hand in our study is open to discussion. One view would consider that the robotic hand is a mere distraction for the participants, that they might best ignore in computing sense of agency. Another view would assume that this robotic hand was perceived as a part of a complete chain linking intention, action, means and outcomes. In the present experiment, participants were explicitly told that they controlled the hand through the sensor glove. In the association phases, they learned a contingency between their finger movement and the robot action, thus creating an internal representation of the expected robot action. In the test phases, we clearly observed that non-homologous robot action influenced SoA over the tone, even when the tone matched the participants’ actions. If participants simply ignored the robot in computing agency, this effect should not have occurred. It is common for voluntary actions to be accompanied by some accessory events, in addition to the goal. Our interest was in how these events influence SoA.

We tested our tracking control hypothesis with implicit, rather than explicit measures of agency. In the agency literature, “judgment of agency” refers to explicit report that an outcome was (or not) caused by our own actions. Explicit and implicit measures can dissociate (e.g. [40–41]). We speculate that explicit judgements may promote focus more strongly on ends or outcomes, while implicit judgements may be more sensitive to means. The explicit judgments of agency commonly used in authorship studies [42–43] are readily biased by cognitive and social factors in including desirability of outcomes [7,44]. The combination of explicit reporting and a social meaningful situation in such studies might lead then to underestimate the contribution of means and tracking control to sense of agency.

Interestingly, Kühn et al. [18] found smaller auditory N1 amplitude for congruent compared to incongruent tones, but did not find any relation between this neural activity and explicit agency judgements. Instead, the P3a amplitude (a marker of attentional processes—Luck, 2005) was reduced in trials where participants explicitly accepted agency. Here, we have used implicit rather explicit measures of SoA [44]. Comparing our results with those of Kühn et al. [18], we may speculate that early brain potentials are a neural correlate of implicit SoA, and are means-sensitive. Conversely, later brain potentials such as P3 may reflect explicit judgements about outcome, and follow a distalized, ideomotor pattern. We did not assess explicit judgments of agency in our experiment. Future work should address the relation between implicit and explicit measures of agency and related brain potentials.

To conclude, many recent studies emphasise the retrospective reconstruction of sense of agency based on processing of outcomes. Our study provides first evidence of an additional mechanism, which resembles continuous updating of predictions, rather than retrospection. In sum, generating a SoA over an outcome, involves an on-line, automatic computation that tracks the sequential stages of the causal chain between intention and outcome. This ongoing predictive computation has reliable and enduring effects on SoA over final outcomes. Several computational difficulties arise in computing SoA. First, the brain must distinguish true instrumental control from mere opportunistic correlation of prior thought and intended outcome. Recent evidence has emphasised that human sense of agency is readily fooled by such correlations [43]. However, tracking control offers one important way of distinguishing true control from correlation. In addition, humans are known to take credit over positive outcomes, even if their actual degree of instrumental control is limited [7]. Tracking intermediate steps in the causal chain could offer a mechanism for mitigating such self-serving biases.

## Supporting Information

S1 File **Figure A. Percentage of correct reproductions after each block type.** The first learning block corresponds to the first block of the experiment. Rc-Tc represents blocks displayed after robot congruent-outcome congruent blocks. Rc-Ti represents blocks displayed after robot congruent-outcome incongruent blocks. Ri-Tc represents blocks displayed after robot incongruent-outcome congruent blocks. Ri-Ti represents blocks displayed after robot incongruent-outcome incongruent blocks. **Figure B. Percentage of correct reproductions after each block type.** The first learning block corresponds to the first block of the experiment. Rc-Tc represents blocks displayed after robot congruent-outcome congruent blocks. Rc-Ti represents blocks displayed after robot congruent-outcome incongruent blocks. Ri-Tc represents blocks displayed after robot incongruent-outcome congruent blocks. Ri-Ti represents blocks displayed after robot incongruent-outcome incongruent blocks.
(DOCX)
