# Supplementary material for: The Sense of Agency as Tracking Control
Source: PLoS One. 2016 Oct 14;11(10):e0163892. doi: 10.1371/journal.pone.0163892 (PMC5065211; doi:10.1371/journal.pone.0163892)
Supplement: S1 File — Figure A. Percentage of correct reproductions after each block type. The first learning block corresponds to the first block of the experiment. Rc-Tc represents blocks displayed after robot congruent-outcome congruent blocks. Rc-Ti represents blocks displayed after robot congruent-outcome incongruent blocks. Ri-Tc represents blocks displayed after robot incongruent-outcome congruent blocks. Ri-Ti represents blocks displayed after robot incongruent-outcome incongruent blocks. Figure B. Percentage of correct reproductions after each block type. The first learning block corresponds to the first block of the experiment. Rc-Tc represents blocks displayed after robot congruent-outcome congruent blocks. Rc-Ti represents blocks displayed after robot congruent-outcome incongruent blocks. Ri-Tc represents blocks displayed after robot incongruent-outcome congruent blocks. Ri-Ti represents blocks displayed after robot incongruent-outcome incongruent blocks. (DOCX) [file pone.0163892.s001.docx]

***Learning phase***

We compared the percentage of correct reproductions between the two tones (400 Hz & 600 Hz). The data were subjected to an arcsine transformation to remove ceiling effects. Repeated measures ANOVAs conducted on transformed data revealed no statistical difference between the correct reproductions associated with each tones (*p* > .3). We then compared the percentage of correct reproductions across blocks. We set up this analysis in such a way as to test specific learning effects, rather than mere repetition effects (see Figure A). The blocks were categorized as follows: 1- the first learning block, i.e. not preceded by any experimental conditions, 2- all the blocks that occurred after the robot congruent-tone congruent condition (RC-TC), 3- all the blocks that occurred after robot congruent-tone incongruent condition (RC-TI), 4- all the blocks that occurred after robot incongruent-tone congruent condition (RI-TC), and 5- all the blocks that occurred after robot incongruent-tone incongruent condition (RI-TI). Again, statistical analyses were conducted on transformed data (arcsine). Repeated measures ANOVAs disclosed a significant main effect of the block categories (F(4,60)=16.302, *p* = .007). Paired comparisons indicated that the percentage of correct reproductions was systematically lower for the first block (81.71 %RC, SD=12.96) than for blocks preceding the experimental conditions (C1: 93.46 %RC, SD=6.30; C2: 93.68 %RC, SD = 6.93; C3: 93.66 %RC, SD=6.84; C4: 93.74 %RC, SD=6.25), all *ps* <= .001. The percentage of correct reproductions did not statistically differ between the other blocks (all *p*s > .3). This result indicated that the six trials in each test phase did not abolish the learning established in the association phase.

Figure A: Percentage of correct reproductions after each block type.

The first learning block corresponds to the first block of the experiment. Rc-Tc represents blocks displayed after robot congruent-outcome congruent blocks. Rc-Ti represents blocks displayed after robot congruent-outcome incongruent blocks. Ri-Tc represents blocks displayed after robot incongruent-outcome congruent blocks. Ri-Ti represents blocks displayed after robot incongruent-outcome incongruent blocks.

As in Experiment 1, we compared the percentage of correct reproductions between the two tones (400 Hz & 600 Hz). A Repeated measures ANOVA conducted on arcsine-transformed data revealed no statistical difference between the correct reproductions associated with each tone (*p* > .7). We then compared the percentage of correct reproductions across the five different block as before. Again, statistical analyses were conducted on arcsine-transformed data. Repeated measure ANOVA indicated that, unlike in Experiment 1, the main effect of block categories was not significant (*p* > .8), suggesting that participants were not differently influenced by conditions in the test phases (see Figure B). This is probably due to the training session that participants did prior to the experiment.

Figure B: Percentage of correct reproductions after each block categories.

The first learning block corresponds to the first block of the experiment. Rc-Tc represents blocks displayed after robot congruent-outcome congruent blocks. Rc-Ti represents blocks displayed after robot congruent-outcome incongruent blocks. Ri-Tc represents blocks displayed after robot incongruent-outcome congruent blocks. Ri-Ti represents blocks displayed after robot incongruent-outcome incongruent blocks.
